# Effects of Nonsurgical Periodontal Therapy on Salivary 8-Hydroxy-Deoxyguanosine Levels and Glycemic Control in Diabetes Mellitus Type 2 Patients

**DOI:** 10.3390/biomedicines10092269

**Published:** 2022-09-13

**Authors:** Jelena Mirnic, Milanko Djuric, Ivana Gusic, Tanja Veljovic, Sasa Cakic, Jasmina Katanic, Karolina Vukoje, Bojana Ramic, Snezana Brkic

**Affiliations:** 1 Department of Dental Medicine, Faculty of Medicine, University of Novi Sad, 21000 Novi Sad, Serbia; 2 Dentistry Clinic of Vojvodina, 21000 Novi Sad, Serbia; 3 Faculty of Dental Medicine, University of Belgrade, 11000 Belgrade, Serbia; 4 Children and Youth Health Care Institute of Vojvodina, Department of Biochemistry, Medical Faculty, University of Novi Sad, 21000 Novi Sad, Serbia; 5 Clinic for Infectious Diseases, Clinical Centre of Vojvodina, Department of Infectious Diseases, Faculty of Medicine, University of Novi Sad, 21000 Novi Sad, Serbia

**Keywords:** diabetes mellitus, periodontal disease/therapy, 8-hydroxy-deoxyguanosine, dental scaling, root planing

## Abstract

Diabetes and periodontitis are complex chronic diseases that are potentially interrelated, as well as associated with oxidative stress. Thus, the aim of the present study was to evaluate the influence of nonsurgical periodontal treatment on salivary 8-hydroxy-deoxyguanosine (8-OHdG) levels and glycemic control in patients suffering from both diabetes mellitus type 2 (DM2) and periodontitis. The study sample included 53 DM2 patients, while 31 systemically healthy patients served as controls. Participants in both groups suffered from periodontitis of comparable severity. Periodontal clinical parameters, namely plaque index (PI), gingival index (GI), papilla bleeding index (PBI), probing pocket depth (PPD), and clinical attachment level (CAL) were recorded, along with salivary 8-OHdG levels and glycated hemoglobin (HbA1c). Levels of 8-OHdG were analyzed by ELISA. All aforementioned parameters were evaluated prior to commencing the study and at 90-day follow-up upon nonsurgical periodontal therapy completion. At baseline, salivary levels of 8-OHdG in DM2 patients were significantly higher (1.17 ng/mL) than those measured for the control group (0.75 ng/mL) and showed significant positive correlation with GI and PPD (*p* < 0.05). Three months after nonsurgical periodontal therapy, the salivary 8-OHdG levels were significantly reduced in DM2 patients (*p* < 0.05). Analysis results also revealed statistically significant changes in all measured clinical parameters between baseline and three-month follow-up in both groups (*p* < 0.05). Upon treatment completion, a decline in the HbA1c level was noted in DM group, but it did not reach statistical significance (*p* > 0.05). It can be concluded that DM2 patients benefit from non-surgical periodontal therapy, as indicated by a marked reduction in their salivary 8-OHdG level and a modest improvement in glycemic control. Short-term clinical benefits noted in the DM group were similar to those observed in the non-diabetic periodontal patients.

## 1. Introduction

The worldwide prevalence of diabetes mellitus (DM) is reaching epidemic proportions, resulting in a greater number of individuals experiencing a wide range of comorbidities that reduce the lifespan and placing a significant burden on the healthcare system [1]. It is estimated that, by 2024, 783 million individuals will be affected by DM worldwide [2].

Presently, it is believed that many of the complications related to DM can be mitigated via better glycemic control. Poor metabolic control has been established as a factor contributing to the risk of chronic complications associated with diabetes. Conversely, reduction in HbA1c was found to yield 20–40-fold reduction in the risk of DM-related death, myocardial infarction, and microvascular complications [3].

Many DM patients suffer from periodontitis and some researchers posit presence of an interacting, complex relationship between these conditions [4]. Available evidence also indicates that DM patients suffering from periodontitis are less likely to establish good glycemic control [5]. Thus, adequate periodontal disease management may be beneficial for DM patients, alongside a healthy diet, exercise, and conventional treatment with hypoglycemic agents and insulin [6].

DM patients that undergo nonsurgical periodontal therapy generally attain improved periodontal status, while research findings regarding its effects on their glycemic status is inconsistent [7,8,9,10,11,12]. A recent meta-analysis of published studies indicated that glycemic control can be improved via periodontal therapy [13], while other authors cautioned that available data are insufficient for drawing definitive conclusions [14].

Oxidative stress plays a major role in the pathogenesis of many systemic and oral diseases. Prior research indicates that it may serve as a link between periodontal disease, and systemic conditions such as diabetes mellitus [15]. Oxidative stress arises due to an imbalance between the production of reactive oxygen species and the antioxidant defense, leading to tissue damage. The generated reactive oxygen species, such as superoxide anions, hydroxyl radicals, and peroxyl radicals, cause damage to many biological molecules (including DNA, lipids, and proteins), whereby their prolonged existence in the body promotes severe tissue damage and cell death [16].

The link between glycemic control in patients with DM2 and periodontal status remains poorly understood [6]. Nonetheless, it is presently assumed that presence of pathogenic bacteria in periodontal tissues triggers cytokine production, as well as the release of acute phase proteins and reactive oxygen species (ROS) that impair insulin sensitivity or action over time [6,17]. This view is supported by the findings reported by Allen et al. [18], indicating that DM2 patients with periodontitis have augmented plasma markers of oxidative stress and significantly lowered β cell function, as well as higher HbA1c and fasting glucose levels, compared to matched DM2 patients without periodontitis. Thus, it is biologically plausible that periodontitis may exacerbate glycaemia in patients with DM2 and that, by reducing circulating cytokine levels and oxidative stress, periodontal treatment may assist in the attainment of more optimal glycemic control [18,19,20,21,22].

Considerable research has recently been carried out on 8-hydroxy-deoxyguanosine (8-OHdG), as its levels in blood and saliva can serve as biomarkers of oxidative DNA damage caused by ROS, allowing the effectiveness of periodontal therapy to be assessed [19,23,24]. Findings yielded by these studies indicate that salivary 8-OHdG concentration is significantly higher in periodontitis patients than in periodontitis-free controls and that the concentration of this marker tends to decrease following periodontal treatment [17,19,25]. However, limited information presently exists on the influence of oxidative stress arising from periodontal lesions on the 8-OHdG concentration in DM2 patients [21,22]. Thus, the goal of the present study was to assess the effects of nonsurgical periodontal therapy on the level of salivary 8-OHdG and glycemic control in DM 2 patients with periodontitis.

## 2. Materials and Methods

### 2.1. Study Sample

The individuals that formed the DM group in our prospective experimental clinical study were recruited from the cohort of 89 DM2 patients that presented to our clinic over a 6-month period. They were referred for periodontal examination by their endocrinologist following a routine examination. Only patients aged 30–70 years that were diagnosed with both DM2 (treated with oral antidiabetic agents) and periodontitis were eligible for inclusion into the DM group.

DM2 patients were excluded from the study if they were taking insulin medication, were regular smokers, were prescribed antibiotics in the three months preceding the study, underwent periodontal treatment within the preceding 6-month period, were pregnant, or suffered from other systemic diseases that could have contributed to periodontitis. Thus, the DM sample comprised of 60 DM2 patients with at least two sites at which clinical attachment (CAL) and probing pocket depth (PPD) of ≥3 mm and ≥4 mm at different teeth were identified, respectively, or PPD ≥ 5 mm was noted at one site [26]. Consequently, as diabetes treatment protocol was modified in four cases, and a further three patients failed to attend the 3-month follow-up appointment, the final DM sample utilized in the analyses consisted of 53 individuals (21 males and 32 females; mean age = 59.2 years).

For the control group, 34 individuals not diagnosed with DM but suffering from periodontitis were recruited from the pool of patients referred to a periodontics specialist by their dentist due to suffering from periodontitis (comparable in severity to the DM group). However, 31 periodontitis patients remained at the end of the study (13 of whom were male and 18 were female; mean age = 57.4 years), as two had to be excluded due to antibiotic use and one failed to complete the study protocol. Prior to commencing any treatment, the study protocol was approved by the local Ethics Committee, and fully adhered to the Declaration of Helsinki. Moreover, all individuals recruited for the DM and the control group gave written informed consent.

### 2.2. Periodontal Examination

Clinical examination involved measurement of plaque index (PI) [27], gingival index (GI) [28], papilla bleeding index (PBI) [29], probing pocket depth (PPD), and clinical attachment level (CAL). PPD and CAL were recorded at four sites (mid-buccal, mesio-buccal, mid-lingual, and disto-lingual) per tooth for all teeth using a Michigan “O” probe with William’s markings.

### 2.3. Laboratory Analysis

Oxidative DNA damage was assessed via 8-OHdG concentrations in saliva. For this purpose, participants provided saliva samples in the morning between 9 and 12 a.m., prior to undergoing clinical periodontal measurements [30]. Patients were instructed to abstain from any food or drink (except water) during the 12 h preceding the visit. Unstimulated salivary samples were collected by patients expectorating into disposable tubes and were immediately centrifuged to remove cell debris (3000× *g* 10 min). The supernatants were stored at −80 °C until required for analysis. The 8-OHdG level in the supernatant was determined using a competitive ELISA kit (Cell Biolabs OxiSelect^TM^, San Diego, CA, USA) in line with the manufacturer’s instructions. Each 8-OHdG sample was assayed in duplicate.

The glycated hemoglobin level served as the indicator of the long-term metabolic DM control. Venous blood samples were taken in the morning, prior to periodontal examination.

### 2.4. Periodontal Therapy

In most cases, nonsurgical periodontal therapy involved two one-hour sessions during which affected teeth were subjected to scaling and root planing (SRP) using an ultrasonic device and Gracey curettes. All patients were also provided with guidelines for maintaining oral hygiene at home and were treated by the same therapist who performed clinical evaluations. All periodontal parameters, saliva, and blood samples were evaluated at baseline and three months after the therapy completion.

### 2.5. Statistical Analysis

All study data was analyzed using the IBM SPSS Statistics 20 for Windows (SPSS, Chicago, IL, USA) commercial software package. The results are presented in the form of absolute (*n*) and descriptive statistics (mean ± standard deviation). Before commencing the study, the sample size was determined using the G*Power software 3.1.9.7 for Windows with the power (1–β) of 0.8 and significance α = 0.05. For the comparison between the experimental and the control group, these criteria indicated that 74 patients (47 + 27) were required, while 30 patients were needed for the pre-post treatment comparison. Due to attrition and exclusion, 53 DM2 patients and 31 controls remained at the end of the study, which meets the specified sample size criteria. The Shapiro–Wilk test was performed to determine if data were normally distributed. The differences in the mean values of clinical, metabolic, and oxidative stress parameters (Shapiro–Wilk *p* < 0.05) between groups at baseline, as well as treatment success evaluation, were assessed using the Mann–Whitney test, and the Wilcoxon test was adopted for pre-post treatment comparison. Chi-squared test was performed for gender, and differences in the means between the two groups at baseline with respect to age (Shapiro Wilk *p* = 0.150) and number of teeth (Shapiro–Wilk *p* = 0.139) were evaluated via student’s *t*-test. Spearman correlation was used to evaluate the relationship between 8-OHdG values and clinical parameters (PI, GI, PBI, PPD, and CAL) as well as HbA1c at baseline. Findings were deemed statistically significant at *p* < 0.05.

## 3. Results

Pertinent characteristics of the DM and the control group are reported in Table 1.

At baseline, relative to the control group, DM2 patients showed statistically significantly higher PI (1.86), GI (1.65), and PBI (1.81) values (PI = 1.32; GI = 0.94, PBI = 1.45), while no statistically significant differences in the PPD and CAL values were observed between the two groups (Table 2).

Three months after completing periodontal therapy, periodontal parameter values in both examined groups declined relative to the baseline and all differences were statistically significant. In terms of the treatment success, the differences between the DM2 patients and the controls in terms of the reduction noted in most clinical parameters were not statistically significant (ΔPI *p* = 0.308; ΔGI *p* = 0.081; ΔPBI *p* = 0.788; ΔCAL *p* = 0.163). In the DM group, PPD reduction was significantly less pronounced compared to that measured for the control group, 0.12 mm vs. 0.34 (*p* = 0.000).

At baseline (Table 3), salivary levels of 8-OHdG in DM2 patients showed significant positive correlation with GI and PPD and were significantly higher (1.17 ng/mL) than those measured for the control group (0.75 ng/mL) (Table 4). Three months after the periodontal treatment, the 8-OHdG levels were lower in both groups, but the reduction was statistically significant in the DM group only (*p* = 0.042).

In DM group, the level of glycemic control did not change significantly during the study (Table 4). Three months after treatment completion, the mean HbA1c decreased by 0.12% in this group, compared to 0.05% recorded for control group. The percentage reduction noted in the HbA1c level for the two groups was not statistically significant.

## 4. Discussion

Initially, significantly higher PI, GI, and PBI values were noted for DM2 patients relative to the controls. These results are consistent with those reported by other authors who noted a higher risk to periodontal disease in DM2 subjects compared with systemically healthy individuals [31,32,33]. In extant research, it is also postulated that diabetes exacerbates inflammatory host response [4]. However, no statistically significant differences were noted between the DM and the control group with respect to CAL and PPD, which are recognized as the main indicators of periodontal damage.

A significant improvement in periodontal status was noted in both DM and control group by the end of the study. A comparison of periodontal healing revealed comparable improvements in plaque and gingival indices, as well as in papilla bleeding index and clinical attachment level. The only difference between the groups pertained to probing pocket depth, as a significantly lower PPD reduction (0.12 mm) was observed in the DM compared to the control group (0.34 mm). This finding was expected, given that the DM group had low PPD at baseline, and thus benefitted less from periodontal treatment [34]. These findings are in line with previously published reports indicating that, in the short term, periodontal treatment yields similar outcomes irrespective of patients’ diabetic status [34,35,36].

Oxidative stress plays a significant role in the pathogenesis of diabetes mellitus, and the available evidence points to a correlation between the severity of diabetes (as indicated by HbA1c levels in blood) and the degree of oxidative stress in serum and saliva [37,38]. Findings reported by several authors also indicate that periodontal disease increases oxidative stress [17,19,39,40]. Specifically, Takane et al. [39] and Konopka et al. [40] respectively measured significantly higher 8-OHdG levels in the saliva and blood of periodontal patients compared to healthy subjects. Pertinent literature further indicates that increased levels of oxidative stress markers in the blood of patients with periodontal disease can negatively affect their systemic health [41]. In addition, results obtained in previous experimental studies conducted on animals suggest that, by exacerbating circulating oxidative stress, periodontitis induces oxidative damage in the liver and descending aorta [42,43,44]. For example, Tomofuji et al. [42] reported oxidative DNA damage in the liver of rats with lipopolysaccharide/protease-induced periodontitis, along with enhanced hydrogen peroxide serum levels. Periodontitis could have a similar effect in patients with diabetes, given that hyperglycemia results in an increased production of free radicals along with a reduced antioxidant protection capacity in the affected individuals [45].

In most extant clinical studies, however, the focus was solely on patients diagnosed with periodontitis, with no underlying medical condition, such as diabetes. Their findings indicate that salivary 8-OHdG levels are related to the degree of periodontal damage [39,46]. For example, Badea et al. [46] measured salivary 8-OHdG in the 5.25–7.50 ng/mL range for participants with the CPITN score ≤ 3, compared to 3.00–5.00 ng/mL for those with scores ≤ 2. Similarly, Takane and colleagues [39] measured statistically significantly higher salivary 8-OHdG levels (4.78 ng/mL) in patients affected by periodontitis who had teeth indicated for extraction compared to patients with no teeth in the terminal periodontitis phase (2.35 ng/mL). These authors posited that 8-OHdG in saliva is primarily associated with significant periodontal tissue damage. They further noted that nonsurgical periodontal therapy can result in a statistically significant reduction in the 8-OHdG levels in patients in whom teeth with poor prognosis are extracted. This is contrasted with the results obtained in patients that had no teeth in the terminal periodontitis phase, in whom 8-OHdG reduction following periodontal treatment was not statistically significant. In comparison to the findings reported in extant studies, our analyses revealed both lower range (0.14–2.0 ng/mL) and mean 8-OHdG values (0.75 ng/mL) in the saliva of patients in the control group, who while suffering from periodontitis did not have DM2. After nonsurgical periodontal treatment salivary 8-OHdG levels declined from 0.75 ng/mL to 0.64 ng/mL in control group, but this difference wasn’t statistically significant. We posit that these results are influenced by a lesser periodontal damage in our patients, due to which very few individuals in control group had teeth in the terminal periodontitis phase. The findings reported here are in line with those obtained by Dede et al. [24], who measured 0.61 ng/mL 8-OHdG concentration in saliva in systemically healthy patients with 2.12 mm periodontal pocket depth. After receiving nonsurgical periodontal treatment, salivary 8-OHdG level declined to 0.53 ng/mL and this difference was not statistically significant.

At baseline, the DM group had significantly higher mean 8-OHdG in saliva (1.17 ng/mL) relative to the controls (0.75 ng/mL), which was probably the result of greater periodontal tissue inflammation in DM group at baseline, as we found a positive significant correlation between 8-OHdG and GI, which is a widely adopted clinical tissue inflammation indicator [47]. Further, salivary 8-OHdG values at baseline were not significantly correlated with HbA1c. Our findings are congruent with those obtained by Dede et al. [48], suggesting that salivary 8-OHdG levels in obese patients are primarily governed by the extent of periodontal inflammation. Su et al. [30] also reported significantly higher mean levels of 8-OHdG in the saliva of DM patients relative to controls, although they neither declared the degree of diabetes metabolic control nor analyzed periodontal status in their work, even though both can affect the reported results. Higher values of 8-OHdG in our DM2 patients at baseline are probably the reason for the statistically significant reduction in mean salivary 8-OHdG measured after nonsurgical therapy, in contrast to the patients of the control group.

In DM patients, periodontal healing was associated with HbA1c reduction from 7.36% to 7.24%, which was not statistically significant, in accordance with extant findings [10,11,12]. For example, Correa et al. [12] reported a non-significant reduction in HbA1c values (from 9.1% to 8.7%) after periodontal therapy in DM2 patients. Conversely, other authors indicated that periodontal infection management improves metabolic control [7,8,9,49,50]. However, in some of these studies, periodontal therapy was provided in addition to antibiotic administration [7,49]. As the effects of scaling and root planing on HbA1c could be masked by systemic antibiotic use, it potentially contributed to the observed reduction in HbA1c levels [10]. Stewart and colleagues [50] noted a reduction in HbA1c levels from 9.5% to 7.6% following periodontal therapy. However, they also reported that, during the study, the oral anti-hyperglycemic medication dose was increased in nearly half of the sample, which hinders objective assessment of the periodontal treatment effectiveness in improving metabolic diabetes control. It is worth noting that a direct comparison of findings reported by different authors is rarely possible due to the differences in study protocols, such as variations in baseline HbA1c or periodontal disease severity, antibiotics use, the follow-up period or sample size, and/or the control group characteristics [10].

As the present study aimed at assessing glycemic control following periodontal therapy, only patients that had no modifications to their diabetic control regimen during the three-month study period were assessed. However, when interpreting our findings, some limitations should be considered. First, the periodontal disease in the patients included in the study sample ranged from mild to severe. As more severe periodontal disease tends to exert a greater influence on hyperglycemia, patients with lower degrees of periodontal damage could not have achieved comparable improvements [51]. Second, as at baseline, glycemic control was not compromised in nearly half of the DM sample, and the effects of periodontal therapy on HbA1c levels was less pronounced [8,51]. A further study limitation stems from the absence of an additional control group comprising of diabetic patients who were not given any periodontal treatment, which could have allowed another level of comparison.

## 5. Conclusions

Despite previously noted study limitations, the findings reported here suggest that DM2 patients benefit from nonsurgical periodontal therapy, as indicated by a marked reduction in their salivary 8-OHdG level and a modest improvement in glycemic control. Future research is thus needed to identify the factors influencing the extent of HbA1c reduction, such as periodontal disease severity and glycemic control at baseline, as well as periodontal treatment and study protocols. Short-term clinical benefits of nonsurgical periodontal therapy were similar in diabetic and non-diabetic periodontal patients.

## Figures and Tables

**Table 1:** Patient characteristics at baseline.

	DM Group (*n* = 53)	Control Group (*n* = 31)	*p*
Gender: Male/Female (*n*)	21/32	13/18	0.507
Age (years) (mean ± SD)	59.23 ± 6.91	57.42 ± 7.33	0.261
Number of teeth (mean ± SD)	17.04 ± 4.81	20.32 ± 5.22	0.004 **
DM duration (years) (mean ± SD)	7.74 ± 5.68		

DM = diabetes mellitus; DM group = DM2 patients; Control group = non-diabetic patients; *n* = number of patients; SD = standard deviation; ** Statisticaly significant difference *p* < 0.01.

**Table 2:** Comparison of periodontal parameters at baseline and at three months upon periodontal therapy completion.

	Group	Baseline	At 3-Month Follow-Up	Change Δ (Baseline−3 Mo)	^a^ *p*	^b^ *p*	^c^ *p*
PI	DM Control	1.86 ± 0.41 1.32 ± 0.51	1.26 ± 0.40 0.66 ± 0.49	0.60 ± 0.35 0.66 ± 0.39	0.000 ***	0.000 *** 0.000 ***	0.308
GI	DM Control	1.65 ± 0.58 0.94 ± 0.72	0.91 ± 0.41 0.37 ± 0.45	0.74 ± 0.45 0.57 ± 0.53	0.000 ***	0.000 *** 0.000 ***	0.081
PBI	DM Control	1.81 ± 0.72 1.45 ± 0.82	1.00 ± 0.59 0.67 ± 0.45	0.81 ± 0.61 0.78 ± 0.54	0.022 *	0.000 *** 0.000 ***	0.788
PPD; mm	DM Control	2.16 ± 0.49 2.38 ± 0.60	2.05 ± 0.46 2.05 ± 0.52	0.12 ± 0.21 0.34 ± 0.23	0.089	0.000 *** 0.000 ***	0.000 ***
CAL; mm	DM Control	2.76 ± 1.24 2.32 ± 1.39	2.51 ± 1.21 1.98 ± 1.31	0.25 ± 0.26 0.34 ± 0.3	0.137	0.000 *** 0.000 ***	0.163

Values are expressed as mean ± SD; Δ—changes in values from baseline to the 3-month follow-up; PI = plaque index; GI = gingival index; PBI = papilla bleeding index; PPD = probing pocket depth; CAL = clinical attachment level; DM group = DM2 patients; Control group = non-diabetic patients; * Statisticaly significant difference *p* < 0.05; *** Statisticaly significant difference *p* < 0.001. ^a^*p* value pertains to differences noted between the groups at baseline (Mann-Whitney test); ^b^*p* value pertains to the longitudinal changes within each group (Wilcoxon test); ^c^*p* value relates to the comparison of changes in parameters between treatment groups (Mann-Whitney test).

**Table 3:** Correlations between 8-OHdG and clinical parameters (PI, GI, PBI, PPD, and CAL) as well as HbA1c at baseline.

	PI	GI	PBI	PPD	CAL	HbA1c
8-OHdG (DM group)	0.087	0.004 **	0.214	0.013 *	0.207	0.161
8-OHdG (Control group)	0.064	0.383	0.088	0.254	0.402	0.847

8-OHdG = 8-hydroxy-deoxyguanosine; PI = plaque index; GI = gingival index; PBI = papilla bleeding index; PPD = probing pocket depth; CAL = clinical attachment level; HbA1c = glycated hemoglobin; DM group = DM2 patients; Control group = non-diabetic patients; * Correlation is significant at the 0.05 level (Spearman correlation). ** Correlation is significant at the 0.01 level (Spearman correlation).

**Table 4:** Comparison of 8-OHdG and HbA1c at baseline and at three months upon periodontal therapy completion.

	Group	Baseline	At 3-Month Follow-Up	Change Δ (Baseline−3 Mo)	^a^ *p*	^b^ *p*	^c^ *p*
8-OHdG (ng/mL)	DM Control	1.17 ± 0.86 0.75 ± 0.54	0.95 ± 0.56 0.64 ± 0.40	0.23 ± 0.56 0.11 ± 0.33	0.036 *	0.042 * 0.088	0.393
HbA1c (%)	DM Control	7.36 ± 1.58 5.51 ± 0.32	7.24 ± 1.49 5.46 ± 0.27	0.12 ± 1.31 0.05 ± 0.15	0.000 ***	0.255 0.051	0.170

Values are expressed as mean ± SD; HbA1c = glycated hemoglobin, 8-OHdG = 8-hydroxy-deoxyguanosine; Δ—changes in values from baseline to the 3-month follow-up; DM group = DM2 patients; Control group = non-diabetic patients; * Statisticaly significant difference *p* < 0.05; *** Statisticaly significant difference *p* < 0.001. ^a^*p* value pertains to differences noted between the groups at baseline (Mann-Whitney test); ^b^*p* value pertains to the longitudinal changes within each group (Wilcoxon test); ^c^*p* value relates to the comparison of changes in parameters between treatment groups (Mann-Whitney test).

## References

[1] Chapple I.L.C., Genco R., On Behalf of Working Group 2 of the Oint EFP/AAP Workshop (2013). Diabetes and periodontal diseases: Consensus report of the joint EFP/AAP workshop on periodontitis and systemic diseases. J. Clin. Periodontol..

[2] Boyko E.J., Magliano D.J., Karuranga S., Piemonte L., Riley P., Saeedi P., Sun H. IDF Diabetes Atlas 2021.

[3] Stratton I.M., Adler A.I., Neil H.A.W., Matthews D.R., Manley S.E., Cull C.A., Hadden D., Turner R.C., Holman R.R. (2000). Association of glycaemia with macrovascular and microvascular complications of type 2 diabetes (UKPDS 35): Prospective observational study. BMJ.

[4] Vitkov L., Muñoz L.E., Knopf J., Schauer C., Oberthaler H., Minnich B., Hannig M., Herrmann M. (2021). Connection between Periodontitis-Induced Low-Grade Endotoxemia and Systemic Diseases: Neutrophils as Protagonists and Targets. Int. J. Mol. Sci..

[5] Costa K.L., Taboza Z.A., Angelino G.B., Silveira V.R., Montenegro R., Haas A.N., Rego R.O. (2017). Influence of Periodontal Disease on Changes of Glycated Hemoglobin Levels in Patients with Type 2 Diabetes Mellitus: A Retrospective Cohort Study. J. Periodontol..

[6] Taylor J.J., Presaw P.M., Lalla E. (2013). A review of the evidence for pathogenic mechanisms that may link periodontitis and diabetes. J. Clin. Periodontol..

[7] Munjal A., Jain Y., Kote S., Krishnan V., Fahim R., Metha S.S., Passi D. (2019). A study on the change in HbA1c levels before and after non-surgical periodontal therapy in type-2 diabetes mellitus in generalized periodontitis. J. Fam. Med. Prim. Care.

[8] Kaur P.K., Narula S.C., Rajput R., Sharma R.K., Tewari S. (2015). Periodontal and glycemic effects of nonsurgical periodontal therapy in patients with type 2 diabetes stratified by baseline HbA1c. J. Oral Sci..

[9] Merchant A.T., Georgantopoulos P., Howe C.J., Virani S.S., Morales D.A., Haddock K.S. (2016). Effect of long-term periodontal care on hemoglobin A1c in type 2 diabetes. J. Dent. Res..

[10] Engebretson S.P., Hyman L.G., Michalowicz B.S., Schoenfeld E.R., Gelato M.C., Hou W., Seaquist E.R., Reddy M.S., Lewis C.E., Oates T.W. (2013). The effect of nonsurgical periodontal therapy on hemoglobin A1c levels in persons with type 2 diabetes and chronic periodontitis: A randomized clinical trial. JAMA.

[11] Auyeung L., Wang P.W., Lin R.T., Hsieh C.J., Lee P.Y., Zhuang Y., Chang H.W. (2012). Evaluation of periodontal status and effectiveness of non-surgical treatment in patients with type 2 diabetes mellitus in Taiwan for a one-year period. J. Periodontol..

[12] Correa F.O.B., Goncalves D., Figueredo C.M.S., Bastos A.S., Gustafsson A., Orrico S.R.P. (2010). Effects of periodontal treatment on metabolic control, systemic inflammation and cytokynes in patients with type 2 diabetes. J. Clin. Periodontol..

[13] Baeza M., Morales A., Cisterna C., Cavalla F., Jara G., Isamitt Y., Pino P., Gamonal J. (2020). Effect of periodontal treatment in patients with periodontitis and diabetes: Systematic review and meta-analysis. J. Appl. Oral Sci..

[14] Faggion C.M., Cullinan M.P., Atieh M. (2016). An overview of systematic reviews on the effectieness of periodontal treatment to improve glycaemic control. J. Periodontal Res..

[15] Li X., Kolltveit K.M., Tronstad L., Olsen I. (2000). Systemic diseases caused by oral infection. Clin. Microbiol. Rev..

[16] Sardi J.O. (2013). Oxidative stress in diabetes and periodontitis. N. Am. J. Med. Sci..

[17] Kurgan S., Önder C., Altingöz S.M., Bagis N., Uyanik M., Serdar M.A., Kantarci A. (2015). High sensitivity detection of salivary 8-hydroxy deoxyguanosine levels in patients with chronic periodontitis. J. Periodontal Res..

[18] Allen E.M., Matthews J.B., O’Halloran D.J., Griffiths H.R., Chapple I.L. (2011). Oxidative and inflammatory status in type 2 diabetes patients with periodontitis. J. Clin. Periodontol..

[19] Önder C., Kurgan S., Altingöz S.M., Bağıs N., Uyanık M., Serdar M.A., Günhan M. (2017). Impact of non-surgical periodontal therapy on saliva and serum levels of markers of oxidative stress. Clin. Oral Investig..

[20] Pendyala G., Thomas B., Joshi S.R. (2013). Evaluation of total antioxidant capacity of saliva in type 2 diabetic patients with and without periodontal disease: A case-control study. N. Am. J. Med. Sci..

[21] Gopalakrishnan S., Ramakrishnan T., Harinath P., Moses J., Shankarram V., Raj S. (2017). Effect of non-surgical periodontal therapy on plasma level of reactive oxygen metabolites and glycemic status in type 2 diabetic patients with chronic periodontitis. Biosci. Biotechnol. Res. Asia.

[22] Muthuraj M.S., Janakiram S., Chithresan K., Maradi A.P., Maddur P.K., Rangaraju R. (2017). Effect of scaling and root planing on levels of 8-hydroxydeoxyguanosine in gingival crevicular fluid of chronic periodontitis patients with and without Type II diabetes mellitus. J. Indian Soc. Periodontol..

[23] Muthuraj M.S.A., Janakiram S., Chithresan K. (2021). Is 8-OHdG a reliable marker in periodontitis—The sixth complication of diabetes mellitus?. Clin. Dent..

[24] Dede F.Ö., Ozden F.O., Avci B. (2013). 8-OHdG levels in gingival crevicular fluid and saliva from patients with chronic periodontitis during initial periodontal treatment. J. Periodontol..

[25] Veljović T., Đurić M., Gušić I., Mirnić J., Čakić S., Maletin A., Brkić S. (2020). The influence of periodontal disease treatment on 8-hydroxy-deoxyguanosine concentrations in saliva and plasma of chronic periodontitis patients. Acta Clin. Croat..

[26] Eke P.I., Page R.C., Wei L., Thornton-Evans G., Genco R.J. (2012). Update of the case definitions for population based surveillance of periodontitis. J. Periodontol..

[27] Silness J., Löe H. (1964). Periodontal disease in pregnancy (II). Correlation between oral hygiene and periodontal condition. Acta Odontol. Scand..

[28] Löe H., Silness P. (1963). Periodontal disease in pregnancy I. Acta Odontol. Scand..

[29] Saxer U., Turconi B., Elsässer C. (1977). Patient motivation with the papillary bleeding index. J. Prev. Dent..

[30] Su H., Velly A.M., Salah M.H., Benarroch M., Trifiro M., Schipper H.M., Gornitsky M. (2012). Altered redox homeostasis in human diabetes saliva. J. Oral Pathol. Med..

[31] Goncalves D., Correa F.O.B., Khalil N.M., de Faria Oliveira O.M.M., Orrico S.R.P. (2008). The effect of non-surgical periodontal therapy on peroxidase activity in diabetic patients: A case-control pilot study. J. Clin. Periodontol..

[32] Serrano C., Perez C., Rodríguez M. (2012). Periodontal conditions in a group of Colombian type 2 diabetic patients with different degrees of metabolic control. Acta Odontol. Latinoam..

[33] Lim L.P., Tay F.B.K., Sum C.F., Thai A.C. (2007). Relationship between markers of metabolic control and inflammation on severity of periodontal disease in patients with diabetes mellitus. J. Clin. Periodontol..

[34] Michalowicz B.S., Hyman L., Hou W., Oates T.W., Reddy M., Paquette D.W., Katancik J.A., Engebretson S.P. (2014). Diabetes and Periodontal Therapy Trial Study Team. Factors associated with the clinical response to nonsurgical periodontal therapy in people with type 2 diabetes mellitus. J. Am. Dent. Assoc..

[35] Mirnić J., Đurić M., Predin T., Gušić I., Petrović Đ., Anđelković A., Bajkin B. (2013). Impact of the level of metabolic control on the non-surgical periodontal therapy outcomes in diabetes mellitus type 2 patients—Clinical effects. Srp. Arh. Celok. Lek..

[36] Mirnić J., Đurić M., Nikolić N., Veljović T., Gušić I., Petrović Đ., Milašin J. (2021). Clinical and microbiological assessment of non-surgical treatment of chronic periodontitis in controlled and uncontrolled type 2 diabetic patients. Acta Clin. Croat..

[37] Memisogullari R., Taysi S., Bakan E., Capoglu I. (2003). Antioxidant status and lipid peroxidation in type II diabetes mellitus. Cell Biochem. Funct..

[38] Kesavulu M.M., Rao B.K., Giri R., Vijya J.S., Subramanyam A.C.H. (2001). Lipid peroxidation and antioxidant enzyme status in type 2 diabetics with coronary heart disease. Diabetes Res. Clin. Pract..

[39] Takane M., Sugano N., Ezawa T., Uchiyama T., Ito K. (2005). A marker of oxidative stress in saliva: Association with periodontally-involved teeth of a hopeless prognosis. J. Oral Sci..

[40] Konopka T., Król K., Kopeć W., Gerber H. (2007). Total antioxidant status and 8-hydroxy-2-deoxyguanosine levels in gingival and peripheral blood of periodontitis patients. Arch. Immunol. Ther. Exp..

[41] Tomofuji T., Irie K., Sanbe T., Azuma T., Ekuni D., Tamaki N., Yamamoto T., Morita M. (2009). Periodontitis and increase in circulating oxidative stress. Jpn. Dent. Sci. Rev..

[42] Tomofuji T., Ekuni D., Yamanaka R., Kusano H., Azuma T., Sanbe T., Tamaki N., Yamamoto T., Watanabe T., Miyauchi M. (2007). Chronic administration of lipopolysaccharide and proteases induces periodontal inflammation and hepatic steatosis in rats. J. Periodontol..

[43] Ekuni D., Tomofuji T., Sanbe T., Irie K., Azuma T., Maruyama T., Tamaki N., Murakami J., Kokeguchi S., Yamamoto T. (2009). Periodontitis-induced lipid peroxidation in rat descending aorta is involved in the initiation of atherosclerosis. J. Periodontal Res..

[44] Ekuni D., Tomofuji T., Tamaki N., Sanbe T., Azuma T., Yamanaka R., Yamamoto T., Watanabe T. (2008). Mechanical stimulation of gingiva reduces plasma 8-OHdG level in rat periodontitis. Arch. Oral Biol..

[45] Kashiwagi A., Asahina T., Ikebuchi M., Tanaka Y., Takagi Y., Nishio Y., Kikkawa R., Shigeta Y. (1994). Abnormal glutathione matabolism and increased cytotoxicity caused by H2O2 in human umbilical vein endothelial cells cultured in high glucose medium. Diabetologia.

[46] Badea V., Balaban D.P., Amariei C., Nuca C., Bucur L. (2010). Salivary 8-hidroxy-2-deoxyguanosine as oxidative stress biomarker for the diagnosis of periodontal disease. Farmacia.

[47] Arana C., Moreno-Fernandez A.M., Gomez-Moreno G., Morales-Portillo C., Serrano-Olmedo I., de la Cuesta Mayor M.C., Hernandez T.M. (2017). Increased salivary oxidative stress parameters in patients with type 2 diabetes: Relation with periodontal disease. Endocrinol. Diabetes Nutr..

[48] Dede F.Ö., Dogan S.B., Balli U., Avci B., Durmuslar M.C. (2016). The effect of initial periodontal treatment on plasma, gingival crevicular fluid and salivary levels of 8-hydroxy-deoxyguanosine in obesity. Arch. Oral Biol..

[49] Amid R., Sovaid M., Saadati H. (2009). Comparison of the effect of non-surgical periodontal therapy with and without systemic doxycycline on the health of periodontium and HbA1c in type 2 diabetic patients without good glicemic control. J. Periodontol. Implant. Dent..

[50] Stewart J.E., Wager K.A., Friedlander A.H., Zadeh H.H. (2001). The effect of periodontal treatment on glycaemic control in patients with type 2 diabetes mellitus. J. Clin. Periodontol..

[51] Chen Y., Zhan Q., Wu C., Yuan Y., Chen W., Yu F., Li Y., Li L.J. (2021). Baseline HbA1c Level Influences the Effect of Periodontal Therapy on Glycemic Control in People with Type 2 Diabetes and Periodontitis: A Systematic Review on Randomized Controlled Trails. Diabetes Ther..

